# Impact of Renin‐Angiotensin System Inhibitors on Renal Function During Temporary Ileostomy Period in Rectal Cancer Patients: A Retrospective Cohort Study

**DOI:** 10.1002/ags3.70122

**Published:** 2025-11-08

**Authors:** Yusaku Shogen, Ryo Seishima, Masayoshi Monno, Satoru Morita, Kohei Shigeta, Koji Okabayashi, Yuko Kitagawa

**Affiliations:** ^1^ Department of Surgery Keio University School of Medicine Tokyo Japan; ^2^ Department of Surgery Fujita Health University Toyoake Aichi Japan; ^3^ Department of Surgery Inagi Municipal Hospital Tokyo Japan

**Keywords:** colorectal neoplasms, ileostomy, proctectomy, renal function, renin‐angiotensin system

## Abstract

**Background:**

This study aims to elucidate the effects of renin‐angiotensin system inhibitors (RASIs) on renal function throughout the stoma period in patients undergoing ileostomy formation and subsequent stoma closure following rectal cancer surgery.

**Methods:**

In this single‐center retrospective study, patients who underwent rectal resection with temporary ileostomy between January 2010 and December 2020 were divided into two groups based on RASI use. Renal function was assessed using the estimated glomerular filtration rate (eGFR) at pre‐surgery (T0), a month post‐surgery (T1), and pre‐stoma closure (T2). The eGFR, its change at each point, and the chronic kidney disease (CKD) classification were used to assess early and subsequent changes in renal function.

**Results:**

Nineteen of 101 patients were using RASI. The RASI and control groups both exhibited eGFR decline at T1, with the former group showing a significantly lower median eGFR (56.2 mL/min/1.73m^2^ vs. 68.7; *p* = 0.007). Although a slight improvement in eGFR was observed, neither group returned to baseline levels by T2. The RASI group showed lower eGFR values at both time points. Multivariate analyses indicated that RASI use was a significant risk factor for renal function impairment in terms of a worse CKD classification at T1 (OR: 9.099; 95% CI: 3.015–27.460; *p* < 0.001).

**Conclusion:**

Our findings suggest that the use of RASIs is associated with early perioperative renal function impairment in patients undergoing ileostomy and stoma closure, with relatively slow recovery. These results indicate the impact of RASIs on eGFR and the importance of careful renal function management.

## Graphical abstract

This retrospective study explored the potential impact of renin–angiotensin system inhibitors (RASIs) on renal function during the ileostomy period in patients undergoing rectal cancer surgery. RASI use was associated with early postoperative renal impairment, and relatively slow recovery of renal function was observed until stoma closure. These findings suggest that careful monitoring of renal function is warranted in patients receiving RASIs after ileostomy.

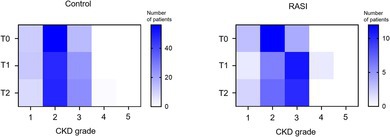

## 1 Introduction

Renal dysfunction after temporary ileostomy formation, particularly when accompanied by rectal cancer surgery, is one of the most common complications faced by patients [1, 2, 3, 4, 5]. This condition poses significant challenges in patient management due to the critical role kidneys play in overall health and recovery. Maintaining renal function is crucial as it can influence the patient's treatment adherence and anti‐cancer drug efficacy, which are essential for adjuvant chemotherapy or the management of recurrence throughout the patient's journey [6, 7].

In the short‐term, high ileostomy output is a well‐documented risk factor for renal dysfunction, leading to significant fluid and electrolyte imbalances that can precipitate acute kidney injury [8, 9]. Additionally, recent studies have identified other risk factors, such as senescence and hypertension, which can further exacerbate renal issues in these patients [10, 11]. These findings underscore the importance of considering patient background when assessing renal function as it is intricately linked with the function of other organs and the overall health status. Therefore, a comprehensive understanding of these risk factors is considered essential for improving patient outcomes.

Recently, there has been increasing attention on the impact of antihypertensive medications on renal function after ileostomy formation. Specifically, renin‐angiotensin system inhibitors (RASIs) have been reported to be associated with dehydration during the acute phase after stoma formation [12, 13]. This dehydration can deteriorate the already delicate fluid balance in patients with ileostomies, consequently leading to readmission. Although some population‐based studies have been conducted, their main outcomes are readmission due to dehydration, and the detailed dynamics of the estimated glomerular filtration rate (eGFR) post‐surgery have not been properly studied. In addition, the effects of RASIs on overall renal function throughout the stoma period remain poorly understood. Therefore, there is a need for more comprehensive research to fully elucidate these impacts.

This study aimed to elucidate the early and subsequent effects of RASIs on renal function throughout stoma period. Understanding the impacts of RASIs on eGFR and renal health could introduce new strategies for monitoring and preserving renal function in patients undergoing ileostomy. This study has the potential to significantly enhance patient care by providing recommendations for the use of antihypertensive drugs in this vulnerable population.

## 2 Materials and Methods

### 2.1 Patients

**FIGURE 1:**
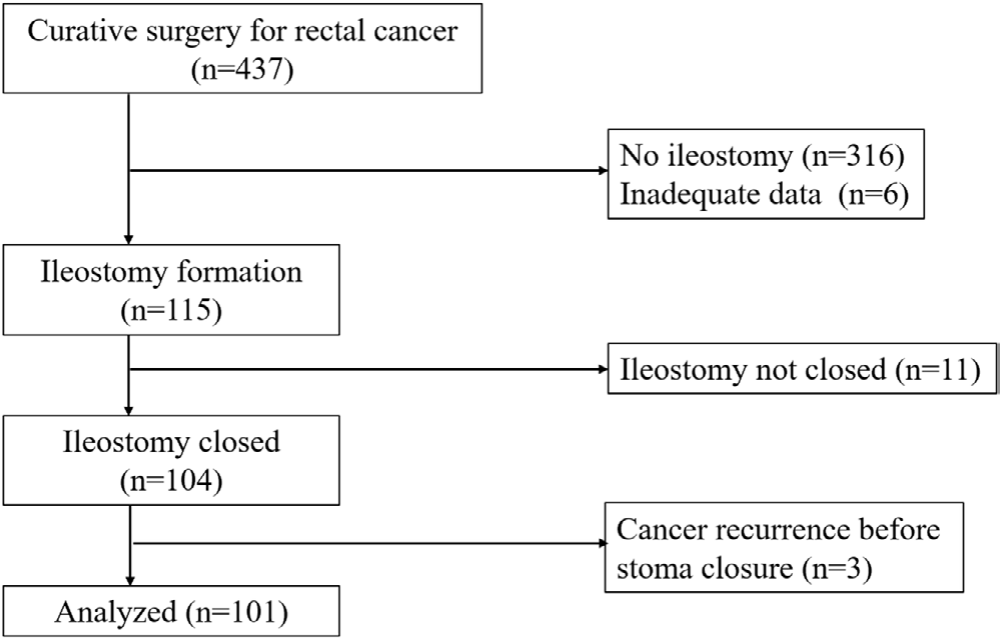
The flow diagram of the study.

This is a retrospective analysis of prospectively collected data from a single institution. We applied the opt‐out method to obtain consent for this study, and the protocol for this research project was approved by a suitably constituted ethics committee of the institution in our hospital (20150051). A total of 437 consecutive patients who underwent curative surgery for rectal cancer at Keio University Hospital from January 2010 to December 2020 were reviewed. As shown in Figure 1, patients who did not have ileostomy (*n* = 316) and those with inadequate data (*n* = 6) were excluded. Of the 115 remaining patients who had ileostomy formation, patients who did not have ileostomy closure during the study period (*n* = 11) and those who had cancer recurrence before stoma closure (*n* = 3) were also excluded. Ultimately, 101 patients were eligible for this study.

### 2.2 Clinicopathological Parameters

Data on following clinicopathological variables were collected: Age, sex, comorbidity, American Society of Anesthesiologists (ASA) score, medication, tumor location, pathological tumor stage, histology, surgical procedure/approach, postoperative complication, neoadjuvant chemoradiotherapy (NACRT), adjuvant chemotherapy, ileostomy duration, ileostomy output at the timing of discharge on the primary surgery, and serum creatinine levels. Serum creatinine values were obtained at three time points: within a month prior to the primary surgery (T0), a month after the primary surgery (T1), and within a month prior to ileostomy closure surgery (T2). The eGFR value was calculated as follows: 194 × Cr (mg/dl)^−1.094^ × age^−0.287^ (male); 194 × Cr (mg/dl)^−1.094^ × age^−0.287^ × 0.739 (female). Postoperative complications were graded according to the Clavien–Dindo grading system, and complications greater than Grade III were counted as a complication. Per the guidelines of the Japanese Society for Cancer of the Colon and Rectum, postoperative adjuvant chemotherapy was recommended for patients with high‐risk pStage II disease (pT4, poorly differentiated histology, less than 12 harvested lymph nodes, diagnosed as bowel obstruction/perforation, or lymphatic/venous invasion) and pStage III disease, and it was performed for 6 months [14, 15]. The regimens used were fluoropyrimidine single (capecitabine or fluorouracil) or doublet (capecitabine + oxaliplatin or fluorouracil + oxaliplatin) [16]. NACRT was performed by the combination of radiation (45Gy/25Fr) and capecitabine (25 days) prior to surgery for patients who have ≥ T3 tumors. As clinical evaluation of renal function, the chronic kidney disease (CKD) grade defined by The Kidney Disease Improving Global Outcomes organization was applied: grade 1 for eGFR ≥ 90, grade 2 for 60–89, grade 3 for 30–59, grade 4 for 15–29, and grade 5 for < 15 [17].

### 2.3 Statistical Analysis

All statistical analyses were performed with Prism 9 (GraphPad Software Inc., CA, USA) or Stata 17 (StataCorp LLC, TX, USA). The Mann–Whitney *U* test and the chi‐square test were applied as appropriate. To evaluate the impact of RASI on renal function, univariate and multivariate analyses were performed using logistic regression models. All *p*‐values were two‐tailed, and the threshold for statistical significance was set at *p* < 0.05.

## 3 Results

### 3.1 Patient Characteristics

The clinicopathological characteristics of the patients are summarized in Table 1. Nineteen (18.8%) out of 101 patients were taking RASIs. The patients taking RASI were significantly older, had a higher ASA score and Charlson Comorbidity Index, and had more diabetes mellitus, compared with the control group. In the control group, 13 patients (15.9%) were taking antihypertensive medications that were not RASIs. Oncologic factors, such as NACRT or adjuvant chemotherapy, did not differ significantly between the two groups. The duration of ileostomy was significantly shorter in the RASI group (median: 111 [98–180] days) than in the control group (median: 152 [113–245] days); *p* = 0.022. Details of concomitant antihypertensive medications, namely calcium channel blockers (CCBs), β blockers, diuretics, are provided in Table S1.

**TABLE 1:** Patients' demographics and clinical characteristics.

		Control (*n* = 82)	RASI (*n* = 19)	*p*
Age (years old)^a^		64.5 (58–71)	72 (64–74)	0.033
Sex	Male	61 (74.4%)	11 (57.9%)	0.152
ASA score	≧ 2	39 (47.6%)	15 (78.9%)	0.013
CCI	≧ 2	7 (8.5%)	6 (31.6%)	0.007
DM	(+)	15 (18.3%)	10 (52.6%)	0.002
Antihypertensive medication	Single	12 (14.6%)	8 (42.1%)	0.007
	Multiple	1 (1.2%)	11 (57.9%)	< 0.001
NACRT	(+)	11 (13.4%)	1 (5.3%)	0.322
Tumor location	Rb	53 (64.6%)	11 (57.9%)	0.583
Distance from AV (cm)^a^		6 (5–10)	7 (6–10)	0.205
Surgical approach	Laparoscopy/Robot	16 (19.5%)	1 (5.3%)	0.184
ISR	(+)	24 (29.3%)	3 (15.8%)	0.388
LLND	(+)	33 (40.2%)	6 (31.6%)	0.485
pStage	3–4	19 (22.0%)	6 (31.6%)	0.556
Histology	Well differentiated	78 (96.3%)	18 (94.7%)	1.000
Postoperative complication	SSI^b^	7 (8.5%)	4 (21.5%)	0.212
	Ileus	4 (4.8%)	1 (5.3%)	1.000
	Others	1 (1.2%)	0	1.000
Adjuvant therapy	Fluoropyrimidine	11 (13.4%)	4 (21.1%)	0.474
	Doublet	7 (8.5%)	2 (10.5%)	0.676
Ileostomy output (mL)^a^		808 (647–1060)	907 (678–1037)	0.812
Ileostomy duration (days)^a^		152 (113–245)	111 (98–180)	0.022

Abbreviations: AL, anastomotic leakage; ASA, American Society of Anesthesiologists; AV, anal verge; CCI, Charlson Comorbidity Index; DM, diabetes mellitus; ISR, intersphincteric resection; LLND, lateral lymph node dissection; NACRT, neoadjuvant chemoradiotherapy; RASI, renin‐angiotensin system inhibitor; SSI, surgical site infection.

^a^ Continuous variables are presented as median (interquartile range).

^b^ SSI includes superficial incisional SSI, deep incisional SSI, and organ/space SSI.

### 3.2 Chronological Change in Renal Function

**FIGURE 2:**
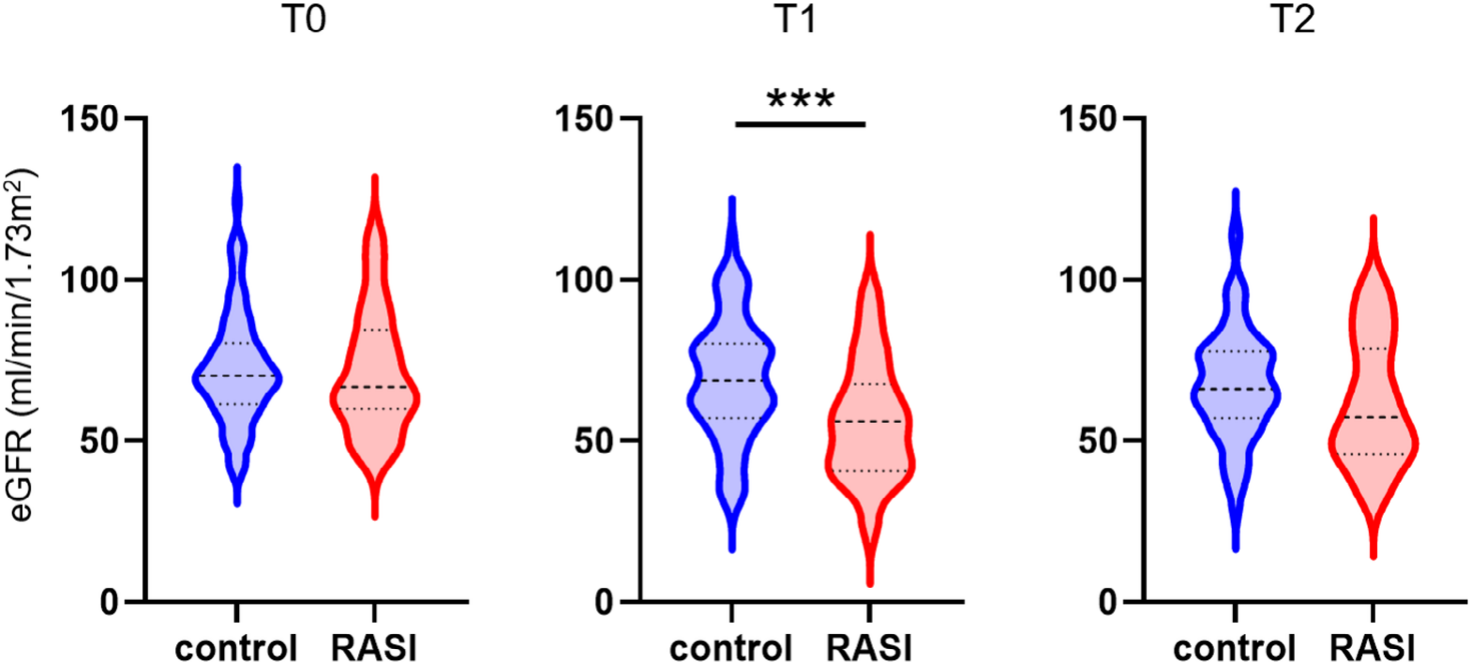
Comparison of eGFR values between the control and RASI groups at different time points. T0, within a month prior to the primary surgery; T1, a month after the primary surgery; T2, within a month prior to ileostomy closure surgery. ***p < 0.01.

To clarify the early and subsequent effects of RASI during the stoma period, we focused on the eGFR for both groups at different time points. Figure 2 illustrates the eGFR comparison at various time points between the two groups. At T0, eGFR did not differ significantly between the groups (median: 70.3 vs. 66.9, *p* = 0.634). However, at T1, the RASI group exhibited a significantly lower eGFR compared with the control group (median: 68.7 vs. 56.2, *p* = 0.007). At T2, the RASI group continued to show relatively lower eGFR values, although the difference was not statistically significant (median 66.2 vs. 57.5, *p* = 0.256).

**FIGURE 3:**
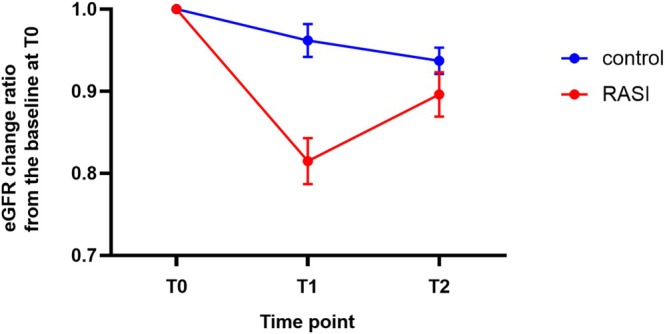
Chronological changes in the eGFR change rate from the baseline at T0. The ratio was calculated by dividing the eGFR at each time point by the eGFR at T0. Each dot and error bar represent the mean ± standard error. T0, within a month prior to the primary surgery; T1, a month after the primary surgery; T2, within a month prior to ileostomy closure surgery.

Given that the baseline eGFR differed between the two groups, albeit not significantly, we went on to evaluate how each patient's eGFR changed after primary surgery, using the rate of change in eGFR based on T0 (Figure 3). Compared with T0, the eGFR in the RASI group decreased to 81.5% at T1, whereas the control group retained to 96.2% (*p* < 0.001). At T2, the RASI group's eGFR recovered to 89.6%, while the control group's eGFR slightly decreased to 92.6%.

### 3.3 Chronological Change in CKD Grade

**FIGURE 4:**
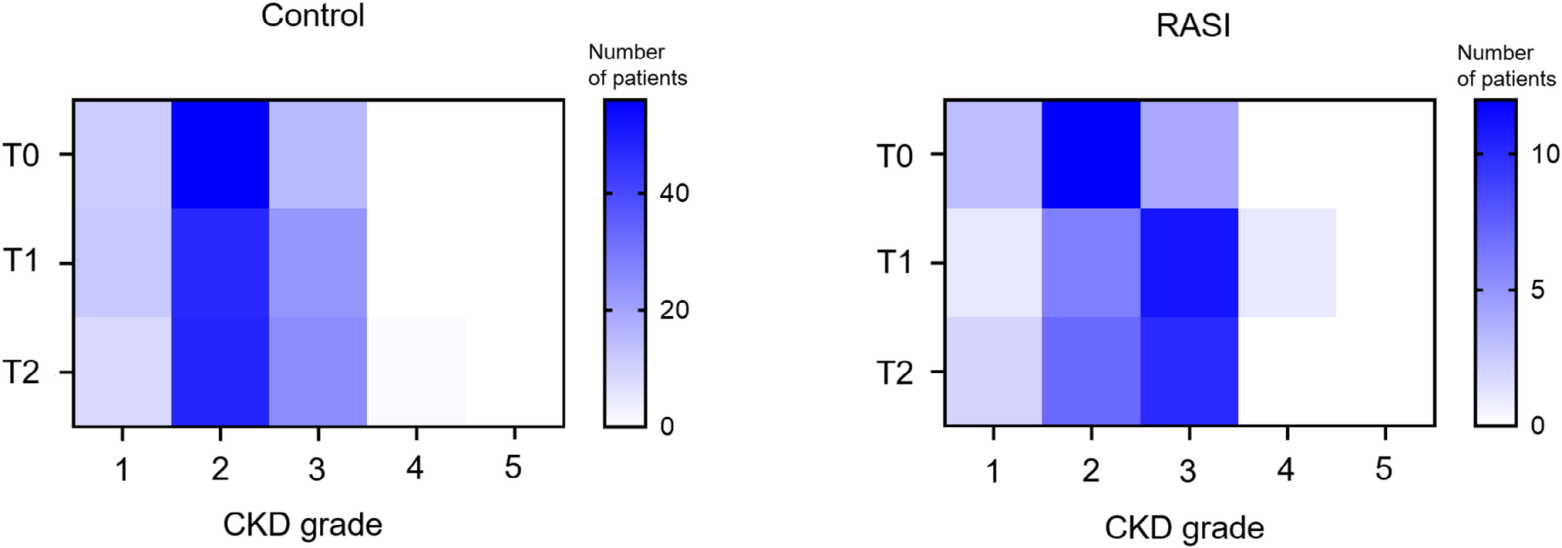
Heatmaps showing chronological changes in the number of patients at each CKD grade in the control and RASI groups. T0, within a month prior to the primary surgery; T1, a month after the primary surgery; T2, within a month prior to ileostomy closure surgery.

Although trends in eGFR over time were found to differ between the RASI and control groups, we used the CKD grade to assess practical utility in clinical practice. Figure 4 shows the chronological changes in CKD grades. At T0, the distribution of CKD grades did not differ significantly between the two groups, with grade 2 being the majority. From T1 onward, the CKD grade shifted toward impairment with grade 3 being the majority in the RASI group, whereas only a slight increase in grade 3 was found in the control group.

### 3.4 Impact of RASIs on Renal Function

**FIGURE 5:**
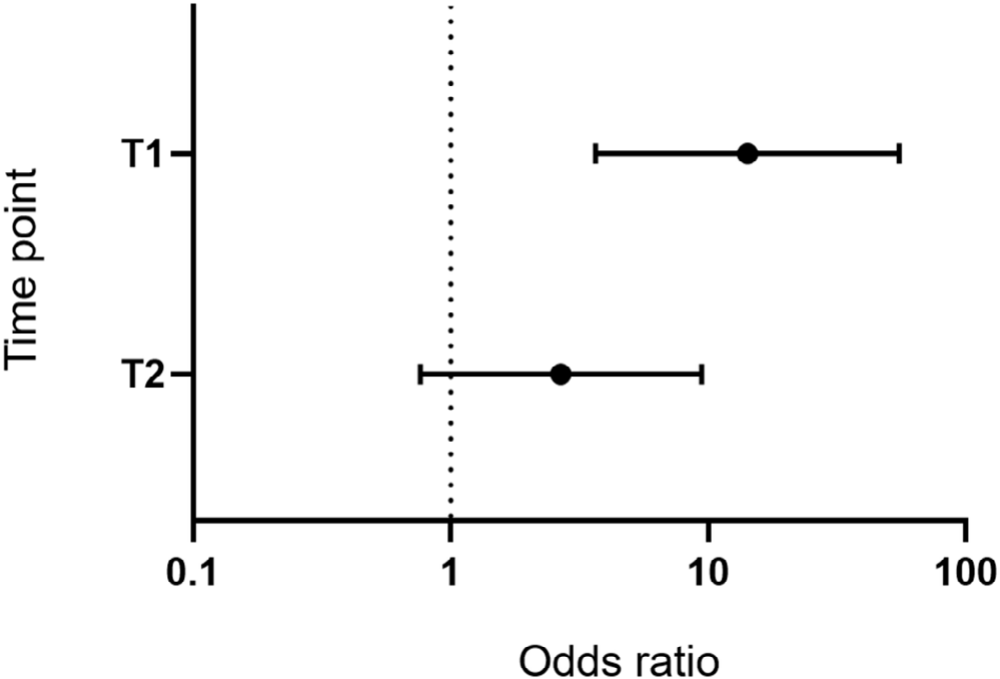
A forest plot showing the impact of RASI on CKD grade changes at each time point, using T0 as the reference. Each dot and error bar represents the odds ratio and 95% confidence interval. T0, within a month prior to the primary surgery; T1, a month after the primary surgery; T2, within a month prior to ileostomy closure surgery.

To evaluate the impact of RASIs on changes in the CKD stage, a multivariate logistic regression analysis was performed, setting the change in the CKD stage from the T0 baseline as the outcome. The results are depicted in Figure 5 as a forest plot; further details are presented in Table S2. Our analysis suggested that RASI use, together with high stoma output, may be independently associated with renal function impairment at the T1 time point. There was also a trend indicating risk of renal function impairment associated with RASI use even in T2, although there was no statistical significance.

### 3.5 Impact of RASIs Compared With Other Antihypertensive Drugs on Renal Function

**FIGURE 6:**
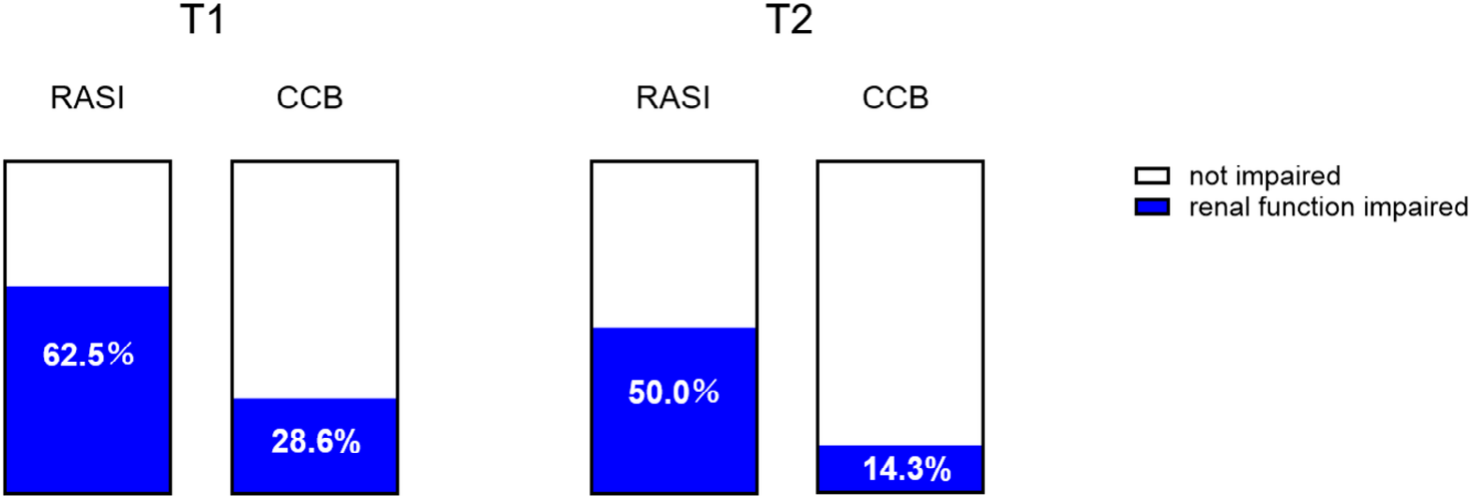
A bar graph comparing the proportion of patients who exhibited renal function impairment during the stoma period between the RASI (*n* = 8) and CCB (*n* = 7) monotherapy groups. CCB, Calcium channel blocker; RASI, renin‐angiotensin system inhibitor; T1, a month after the primary surgery; T2, within a month prior to ileostomy closure surgery.

To evaluate the impact of RASIs compared with other antihypertensive agents on both early and subsequent changes in renal function during the stoma period, we constructed models for the other antihypertensive agents using the same adjustment factor as in the previously presented multivariate analysis. Similar to RASIs, CCBs were significantly associated with renal function deterioration at T1 but not at T2 (Table S3). We next conducted a direct comparison between RASI and CCB by examining the proportion of patients who exhibited renal function impairment among those taking each medication alone (Figure 6). At both T1 and T2, renal function impairment was more frequent in the RASI group than in the CCB group (T1: 62.5% vs. 28.6%; T2: 50.0% vs. 14.3%). Although the differences were not statistically significant, the results suggested a higher risk associated with RASI use (T1: *p* = 0.189; T2: *p* = 0.143).

## 4 Discussion

This study aimed to elucidate the early and subsequent effects of RASI on renal function throughout the stoma period in patients who underwent temporary ileostomy formation and subsequent stoma closure following rectal cancer surgery. Our findings indicate that RASI use is associated with early renal function impairment, as evidenced by a significant reduction in eGFR a month after surgery. Although the subsequent impairment appears to be less pronounced, it still remains noteworthy compared with the control group. Given the relatively small sample size, these results should be interpreted with caution, yet they have important implications for the management of hypertensive patients undergoing ileostomy.

The risk of renal function impairment in the early postoperative period is well‐documented, with a high stoma output and senescence being significant risk factors [1, 18]. In our study, a high stoma output was consistently associated with renal function decline at the T1 time point (Table S2). This underscores the importance of careful postoperative management, especially stoma output monitoring. In addition, our analysis suggested that RASI use represents an independent risk factor for renal impairment, irrespective of stoma output (Table S2). These findings suggest that, in patients receiving RASI therapy, not only careful stoma output monitoring, but also consideration of switching from RASI to alternative antihypertensive agents may be warranted. Adjuvant chemotherapy, which has also been identified as a risk factor for renal function decline, did not significantly impact our results [7]. This may be due to the limited use of oxaliplatin, a nephrotoxic chemotherapeutic agent, within our study cohort.

While early postoperative changes in renal function after stoma formation has been described, prolonged changes during the stoma period have been less extensively studied. A previous study by Yaegashi et al. reported a marked decline in renal function within the first month after stoma formation, followed by stabilization with no significant improvement after stoma closure [9]. Our findings align with these observations, demonstrating a clear decline in eGFR during the first postoperative month. In the RASI group, renal function showed a slight improvement from 1 month after surgery to before stoma closure; however, it remained lower than the baseline eGFR. Moreover, the RASI group had a higher CKD grade even before stoma closure, highlighting the possibility of a subsequent effect. Since prolonged renal function decline leads to an increased risk of cerebrovascular and cardiovascular disease, renal function should be protected as much as possible [19]. We need to keep in mind that RASI use in ileostomy patients would increase those risks; thus, it is preferable to switch to other drugs if possible.

We also considered the potential impact of switching antihypertensive agents from RASIs. As shown in the comparison between RASIs and other antihypertensive agents, neither diuretics nor β‐blockers were significantly associated with renal function changes. Both may therefore serve as potential alternatives to RASIs. However, based on previous reports, diuretics may not be appropriate substitutes [11, 12]. In contrast, CCBs showed results similar to those of RASIs. It should be noted, however, that a considerable number of patients were taking both agents concurrently. In the analysis limited to monotherapy, the risk associated with CCBs seemed to be relatively low, suggesting that CCBs could also be considered potential alternatives. Nonetheless, due to the limited sample size, definitive conclusions cannot be drawn, and further investigations are warranted.

The mechanism by which RASI adversely affects renal function warrants further discussion. RASIs are known to induce a state of reduced intraglomerular pressure by inhibiting angiotensin II, a potent vasoconstrictor of the efferent arterioles [20]. While this mechanism is beneficial in reducing proteinuria and slowing CKD progression in hypertensive and diabetic patients, it may lead to acute reductions in eGFR, particularly in the context of reduced renal perfusion. Therefore, RASIs can exacerbate dehydration following ileostomy, a condition considered to predispose patients to dehydration, promoting further renal function impairment. These impacts highlight the need for a nuanced approach to hypertension management in patients with ileostomies, balancing the protective renal effects of RASI against their potential to cause acute kidney injury in the perioperative period. RASI has recently been reported to have anti‐cancer properties, and it is expected to be applied in patients with colorectal cancer [21, 22]. However, this is a double‐edged blade considering the risk of reduced chemotherapy regimen options due to renal function compromise in the context of ileostomy‐formatted rectal cancer patients. Thus, it is necessary for us to understand the characteristics of this drug and make a decision to use it.

Nevertheless, our study had certain limitations, the first of which is its retrospective design which inherently comes with the risk of selection bias and limits its ability to establish causality. Additionally, our sample size—particularly the number of patients in the RASI group—was relatively small. This may limit the generalizability of our findings and the statistical power to detect significant differences. In particular, baseline differences such as age, comorbidities, and ASA score are factors indicative of elderly patients with reduced physiological reserve. Regardless of RASI use, these factors may have contributed to delayed postoperative renal function recovery. However, since their impacts on renal function were not significant in the univariate analysis, they were not included in the multivariate analysis in this particular study. Future prospective studies with larger cohorts are needed to confirm our findings and further explore the mechanisms underlying RASI‐related renal function impairment. Another limitation is the lack of detailed data on the types and dosages of RASI and other antihypertensive medications used, which could influence renal outcomes.

In conclusion, although exploratory in scope, our study suggests the importance of careful renal function management in patients undergoing ileostomy and stoma closure, particularly those treated with RASI. While these medications are crucial for managing hypertension and protecting against CKD progression, they may pose risks for acute renal impairment and eGFR recovery delay in the stoma period. Our findings underscore the importance of close monitoring and tailored management strategies for these patients.

## Author Contributions

**Yusaku Shogen:** writing – original draft, conceptualization, methodology, data curation, investigation, validation, formal analysis, visualization. **Ryo Seishima:** conceptualization, methodology, data curation, investigation, validation, formal analysis, supervision, visualization, project administration, resources, writing – review and editing. **Masayoshi Monno:** supervision, writing – review and editing, resources. **Satoru Morita:** supervision, resources, writing – review and editing. **Kohei Shigeta:** supervision, resources, writing – review and editing. **Koji Okabayashi:** supervision, resources, writing – review and editing. **Yuko Kitagawa:** writing – review and editing, supervision.

## Ethics Statement

This study was conducted in accordance with the principles of the Declaration of Helsinki. This study was approved by the Ethics Committee of Keio University School of Medicine (Approval No. 20150051). Informed consent for publication was obtained via the opt‐out method, as approved by the ethics committee.

## Conflicts of Interest

Y.K. is the Editor‐in‐Chief of Annals of Gastroenterological Surgery.

## Supporting information


**Table S1:** Details of oral antihypertensive medications.

**Table S2:** Logistic regression analysis of RASI and CKD grade change.

**Table S3:** Logistic regression analysis of each antihypertensive agent and CKD grade change.
