# Supplementary material for: Impact of Renin‐Angiotensin System Inhibitors on Renal Function During Temporary Ileostomy Period in Rectal Cancer Patients: A Retrospective Cohort Study
Source: Ann Gastroenterol Surg. 2025 Nov 8;10(2):527–33. doi: 10.1002/ags3.70122 (PMC12962035; doi:10.1002/ags3.70122)
Supplement: Supplementary file 1 — Table S1: Details of oral antihypertensive medications. [file AGS3-10-527-s003.docx]

**Supplemental Table S1**

Details of oral antihypertensive medications.

|  | Control | RASI | P |
| --- | --- | --- | --- |
|  | (n=82) | (n=19) |  |
| CCB | 8 (9.8%) | 9 (47.4%) | <0.001 |
| β blocker | 3 (3.7%) | 1 (5.3%) | 0.572 |
| Diuretics | 3 (3.7%) | 1 (5.3%) | 0.572 |

| Control Group | (n=82) |
| --- | --- |
| No antihypertensives | 69 (84.1%) |
| CCB | 8 (9.8%) |
| β blocker | 2 (2.4%) |
| Diuretics | 2 (2.4%) |
| β blocker + Diuretics | 1 (1.2%) |

| RASI Group | (n=19) |
| --- | --- |
| RASI only | 8 (42.1%) |
| RASI + CCB | 9 (47.4%) |
| RASI + β blocker | 1 (5.3%) |
| RASI + Diuretics | 1 (5.3%) |

Abbreviations: CCB, Calcium channel blocker; RASI, renin-angiotensin system inhibitor.
