# Supplementary material for: Impact of Renin‐Angiotensin System Inhibitors on Renal Function During Temporary Ileostomy Period in Rectal Cancer Patients: A Retrospective Cohort Study
Source: Ann Gastroenterol Surg. 2025 Nov 8;10(2):527–33. doi: 10.1002/ags3.70122 (PMC12962035; doi:10.1002/ags3.70122)
Supplement: Supplementary file 2 — Table S2: Logistic regression analysis of RASI and CKD grade change. [file AGS3-10-527-s001.docx]

**Supplemental Table S2**

Results of logistic regression analysis assessing the impact of RASI on CKD grade change at different time points.

| T1 | Univariate analysis | | | Multivariate analysis | | |
| --- | --- | --- | --- | --- | --- | --- |
|  | Coefficient | 95% CI | P value | Coefficient | 95% CI | P value |
| Age | 1.016 | 0.972-1.061 | 0.490 |  |  |  |
| Sex | 0.955 | 0.350-2.605 | 0.928 |  |  |  |
| ASA | 0.981 | 0.433-2.224 | 0.964 |  |  |  |
| CCI | 1.283 | 0.804-2.047 | 0.297 |  |  |  |
| Stoma duration | 1.000 | 0.995-1.004 | 0.870 |  |  |  |
| **Stoma output** | **1.002** | **1.000-1.003** | **0.029** | **1.002** | **1.000-1.004** | **0.012** |
| NACRT | 1.015 | 0.252-4.086 | 0.983 |  |  |  |
| Adjuvant (any regimen) | 1.350 | 0.484-3.767 | 0.567 |  |  |  |
| Adjuvant (doublet) | 0.857 | 0.166-4.422 | 0.854 |  |  |  |
| Tumor location | 1.037 | 0.405-2.656 | 0.940 |  |  |  |
| **RASI** | **9.099** | **3.015-27.460** | **< 0.001** | **14.186** | **3.656-55.040** | **< 0.001** |

| T2 | Univariate analysis | | | Multivariate analysis | | |
| --- | --- | --- | --- | --- | --- | --- |
|  | Coefficient | 95% CI | P value | Coefficient | 95% CI | P value |
| Age | 0.992 | 0.951-1.034 | 0.696 |  |  |  |
| Sex | 0.389 | 0.12-1.254 | 0.114 |  |  |  |
| ASA | 0.981 | 0.433-2.224 | 0.964 |  |  |  |
| CCI | 1.142 | 0.709-1.841 | 0.585 |  |  |  |
| Stoma duration | 1.002 | 0.998-1.006 | 0.384 |  |  |  |
| **Stoma output** | **1.002** | **1.000-1.003** | **0.012** | **1.002** | **1.000-1.004** | **0.010** |
| NACRT | 0.574 | 0.117-2.816 | 0.494 |  |  |  |
| Adjuvant (any regimen) | 2.288 | 0.847-6.175 | 0.102 |  |  |  |
| Adjuvant (doublet) | 1.591 | 0.367-6.894 | 0.535 |  |  |  |
| Tumor location | 1.311 | 0.502-3.422 | 0.580 |  |  |  |
| **RASI** | **2.074** | **0.712-6.039** | **0.181** | **2.680** | **0.763-9.413** | **0.124** |

ASA, American Society of Anesthesiologists; CCI, Charlson Comorbidity Index; NACRT, neoadjuvant chemoradiotherapy; RASI, renin-angiotensin system inhibitor; CI, confidence interval
