# Supplementary material for: Impact of Renin‐Angiotensin System Inhibitors on Renal Function During Temporary Ileostomy Period in Rectal Cancer Patients: A Retrospective Cohort Study
Source: Ann Gastroenterol Surg. 2025 Nov 8;10(2):527–33. doi: 10.1002/ags3.70122 (PMC12962035; doi:10.1002/ags3.70122)
Supplement: Supplementary file 3 — Table S3: Logistic regression analysis of each antihypertensive agent and CKD grade change. [file AGS3-10-527-s002.docx]

**Supplemental Table S3**

Results of logistic regression analysis assessing the impact of each antihypertensive agent on CKD grade change at different time points.

| T1 | Multivariate analysis ^†^ | | |
| --- | --- | --- | --- |
|  | OR | 95% CI | P value |
| RASI | 14.186 | 3.656-55.04 | <0.001 |
| CCB | 5.384 | 1.519-19.082 | 0.009 |
| β blocker ^‡^ | - | - | - |
| Diuretics | 6.314 | 0.519-76.778 | 0.148 |

| T2 | Multivariate analysis ^†^ | | |
| --- | --- | --- | --- |
|  | OR | 95% CI | P value |
| RASI | 2.680 | 0.763-9.413 | 0.124 |
| CCB | 2.222 | 0.598-8.253 | 0.233 |
| β blocker | 1.339 | 0.121-14.816 | 0.812 |
| Diuretics | 8.420 | 0.669-105.965 | 0.099 |

† Adjusted for stoma output (mL).

‡The drug administration group had no events; therefore, it was excluded from the logistic regression model

Abbreviations: CCB, Calcium channel blocker; CI, confidence interval; OR, Odds ratio; RASI, renin-angiotensin system inhibitor.
